# Fragmentation of the large subunit ribosomal RNA gene in oyster mitochondrial genomes

**DOI:** 10.1186/1471-2164-11-485

**Published:** 2010-09-02

**Authors:** Coren A Milbury, Jung C Lee, Jamie J Cannone, Patrick M Gaffney, Robin R Gutell

**Affiliations:** 1 College of Earth, Ocean, and Environment, University of Delaware, Lewes, DE, USA; 2 Center for Computational Biology and Bioinformatics, The Institute for Cellular and Molecular Biology, University of Texas, Austin, TX, USA; 3 Dana-Farber Cancer Institute, Harvard Medical School, Boston, MA, USA

## Abstract

**Background:**

Discontinuous genes have been observed in bacteria, archaea, and eukaryotic nuclei, mitochondria and chloroplasts. Gene discontinuity occurs in multiple forms: the two most frequent forms result from introns that are spliced out of the RNA and the resulting exons are spliced together to form a single transcript, and fragmented gene transcripts that are not covalently attached post-transcriptionally. Within the past few years, fragmented ribosomal RNA (rRNA) genes have been discovered in bilateral metazoan mitochondria, all within a group of related oysters.

**Results:**

In this study, we have characterized this fragmentation with comparative analysis and experimentation. We present secondary structures, modeled using comparative sequence analysis of the discontinuous mitochondrial large subunit rRNA genes of the cupped oysters *C. virginica, C. gigas*, and *C. hongkongensis*. Comparative structure models for the large subunit rRNA in each of the three oyster species are generally similar to those for other bilateral metazoans. We also used RT-PCR and analyzed ESTs to determine if the two fragmented LSU rRNAs are spliced together. The two segments are transcribed separately, and not spliced together although they still form functional rRNAs and ribosomes.

**Conclusions:**

Although many examples of discontinuous ribosomal genes have been documented in bacteria and archaea, as well as the nuclei, chloroplasts, and mitochondria of eukaryotes, oysters are some of the first characterized examples of fragmented bilateral animal mitochondrial rRNA genes. The secondary structures of the oyster LSU rRNA fragments have been predicted on the basis of previous comparative metazoan mitochondrial LSU rRNA structure models.

## Background

The translation of mRNA into functional proteins occurs within the two subunits of the ribosome. These subunits are typically 30S and 50S (to form the 70S ribosome) in prokaryotes and chloroplasts, 40S and 60S (to form the 80S ribosome) in nuclear encoded eukaryotic ribosomes, and subunit sizes vary considerably in mitochondria.

The small ribosomal subunit (SSU) typically contains a single RNA and exhibits a range of sizes; ribosomal RNA (rRNA) are 16S in prokaryotes and chloroplasts [1], 18S in the eukaryotic nuclear genome [1], and varies from 9S [2] to over 18S in mitochondrial genomes [3]. The RNA in the small ribosomal subunit (30S or 40S) is also called 16S-like rRNA and more typically SSU rRNA. The large ribosomal subunit (LSU) usually contains two or three primary RNAs. Prokaryotic and chloroplast LSUs usually possess a 23S rRNA [1], eukaryotic nuclear genomes contain 25-28S and 5.8S rRNAs [1], and in mitochondria, rRNAs range from 12S to >26S [2,4]. This RNA in the large ribosomal subunit (50S or 60S) is also called 23S-like rRNA and more typically LSU rRNA. The majority of the mitochondrial ribosomes, including animals, do not contain a 5S rRNA in their LSU; the exceptions are plants [5], protists including some red, green, and brown algae [6-15], and more recently an amoeboid protist, *Acanthamoeba castellanii *[16] that contain an RNA with the major characteristics of 5S rRNAs. The functional ribosome with the 30S/40S and 50S/60S subunits contain at least 50 ribosomal proteins (rProteins) [1]. Although phylogenetic variation in size, sequence, and structure of rRNAs is extensive [17], specific regions of the rRNA are conserved in all known organisms. Several of the conserved regions of the SSU and LSU rRNA assemble to form the catalytic core within the center of the ribosome [18].

Typically, 5S, 16S and 16S-like, and 23S and 23S-like rRNAs are each encoded from a continuous rRNA gene; however, the rRNA genes infrequently have non-rRNA nucleotide sequences inserted. The two primary types of interrupted rRNA genes are the result of: 1) introns and 2) fragmentation. Introns, the nucleotides within the rRNA gene that do not code for rRNA, are removed during transcript processing, followed by exon splicing or re-ligating, the parts of the rRNA gene that codes for the rRNA [19]. At least 3000 introns have been identified in rRNA [17,20]. Discontinuous and fragmented rRNAs can be encoded by at least two noncontiguous regions of the genome that when combined code for a full length rRNA. However, unlike introns, the fragments of the rRNA are not covalently reconnected. Thus, the rRNA in the ribosome is split into at least two fragments.

While the majority of the introns in rRNA occur within highly conserved regions of the rRNA gene, fragmentation occurs in regions of high variability [17,20]. In three dimensional space the introns occur on the interface side of the 30S and 50S ribosomal subunits that have a high percentage of very conserved nucleotides while the regions with large insertions and deletions generally occur on the back side of each of the two subunits where the majority of the large insertions occur in organisms that span the entire tree of life [20].

Two modes of gene organization have been observed for the discontinuous rRNA genes. The first maintains the order of the rRNA sequence in the rRNA gene and the intervening nucleotides in the gene are removed after they are transcribed. The regions that are excised are called internal transcribed spacers (ITS) in eukaryotes, and intervening sequences (IVS) in prokaryotes. Although most rRNAs are coded by a contiguous gene, many examples of discontinuous coding within the rRNA have been documented. Within some closely related bacteria of the same genus, one species (or strain) may have an insertion and discontinuous coding at a specific location in the rRNA structure (e.g. *Campylobacter jejuni *[21]) while another species in the same genus does not have an insertion at the same location in the LSU rRNA and has a single continuous 23S rRNA (e.g. *Campylobacter coli *[22]). Many other examples of discontinuity that maintains the genetic order of the functional rRNA have been identified in eukaryotes and have been reviewed in Gray and Schnare [23,24].

The second type of discontinuous genes occurs less frequently. Here the rRNA-encoding regions are separated by tRNA- or protein-encoding genes rather than introns. In a few cases, the functional order of rRNA fragments has been rearranged in addition to the insertion of non-rRNA gene fragments. For example, gene fragmentation and rearrangement have been observed in the SSU and/or LSU rRNAs in the mitochondrial genomes of the fungus *Haloraphidium curvatum *[25], as well as ciliates *Tetrahymena pyriformis *and *Paramecium aurelia *[26,27]. Extensive ribosomal fragmentation and rearrangement have also been observed in rRNA genes in the mitochondrial genomes of chlorophycean and prasionophycean green algae *Chlamydomonas reinhardtii *[28], *C. eugametos *[29], and inferred in other *Chlamydomonas *species [30] and other green algae mitochondria based on isolation of rRNA fragments [31], such as *Chlorogonium elongatum *[32] and *Pedinomonas minor *[7]. The most extensive fragment rearrangement of the rRNA gene occurs in protistan apicomplexans (such as *Theileria parva *[33] and *Plasmodium falciparum *[34-36]) and dinoflagellates [37-39] (such as *Oxyrrhis marina *[40] and *Alexandrium catenella *[41]).

While fragmented and rearranged ribosomal RNA genes have been observed in bacteria and eukaryotic nuclear, chloroplast and mitochondrial RNA genes, the first discontinuous ribosomal RNA gene in a bilateral metazoan mitochondrial genome was reported in 2005 - *Crassostrea virginica *[1] and subsequently found in two other strains of *Crassostrea, C. gigas *[42] and *C. hongkongensis *[43,44] The first discontinuous rRNA gene in a non-bilateral metazoan Placozoan, *Trichoplax adhaerens *was reported in 2006 [45] followed by the identification of three additional discontinuous rRNAs in the same Placozoan phylogenetic group [46].

The characterization and modeling of the secondary structures of the fragmented LSU rRNA genes is essential to determine its location in the rRNA's higher-order structure, gain an understanding of how these gene fragments associate to form functional rRNAs and ribosomes, and relate the conserved and variable regions with the high resolution crystal structures and the functional components of the ribosomal RNA in protein synthesis. This fragmentation is also important as it could help us understand the rearrangements within an organism's genome.

Using reverse-transcriptase polymerase chain reaction (RT-PCR), and analysis of EST sequences, we demonstrate that the two LSU rRNA fragments predicted with comparative analysis are not spliced together post-transcriptionally in *Crassostrea virginica*. The secondary structure of these fragmented ribosomal LSU RNA genes in the cupped oysters *Crassostrea virginica, C. gigas*, and *C. hongkongensis*, have been modeled and are described herein.

## Results and discussion

### RT-PCR analysis of *Crassostrea virginica*

**Figure 1:**
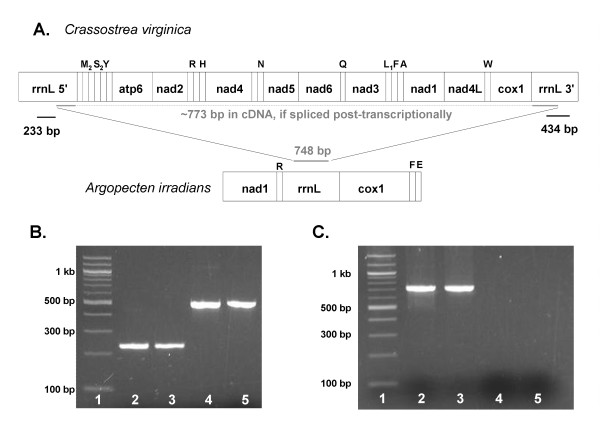
**Amplification of mtDNA and cDNA LSU rRNA gene regions in *Crassostrea virginica***. Diagram A presents the amplicon locations in the *C. virginica *and *A. irradians *mtDNA genome. Gel image B contains a 233 bp region of the 5'-half LSU amplified from the *C. virginica *genomic DNA preparation (lane 2) and cDNA preparation (lane 3), as well as a 494 bp region of the 3'-half LSU amplified from the *C. virginica *genomic DNA preparation (lane 4) and cDNA preparation (lane 5); lane 1 = 100 bp ladder. Gel image C: a 748 bp product (starting in the 5' half and ending in the 3' half) amplified from a continuous LSU template in *Argopecten irradians *genomic DNA (lane 2) and cDNA (lane 3), but was not amplified from *C. virginica *genomic DNA (lane 4) or cDNA (lane 5).

Ribonucleic acid (RNA) and deoxyribonucleic acid (DNA) were individually extracted from adductor muscle of the oyster *Crassostrea virginica*. Reverse-transcriptase (RT)-PCR was performed on the *C. virginica *RNA to generate complementary DNA (cDNA). PCR amplification was performed to amplify portions of the 5' half and 3' halves of the LSU rRNA genes from both the DNA and cDNA templates. The anticipated amplicons were produced for both portions of the LSU rRNA (233 bp amplicon and 434 bp amplicon) from both the DNA and the cDNA template (Table 1, Figure 1). However, a ~773 bp region could not be amplified from the cDNA template using primers located in the 5' and 3' portions of the LSU rRNA gene. Amplification was simultaneously performed in the bay scallop *Argopecten irradians*, a related mollusk with a full length, contiguous LSU gene [47] with regions of conservation where primers were situated (GenBank: DQ665851). As apparent in Figure 1, the 748 bp region of the LSU rRNA gene was successfully amplified in the genomic DNA and cDNA of *Arogopecten irradians*; this region could not be amplified in *Crassostrea virginica *genomic DNA or cDNA. The lack of amplification with the third primer set indicates that *C. virginica *mRNA is not post-transcriptionally modified by splicing to generate a full-length LSU rRNA, nor is there a functional full-length LSU gene elsewhere in the genomic DNA of *C. virginica*.

**Table 1:** Primers used to amplify specific regions of the LSU rRNA gene from oyster (*Crassostrea virginica*) genomic DNA and cDNA template (* primers are from [42]).

Region	Primer	5' to 3' oligonucleotide sequence	Product length	Annealing temperature
5' portion	mt168-F *	GGATTCTGTTTGTCCGCAGCATT	233 bp	50°C

	mt169-R *	CACCATATAGCTATCTTTAGTTGA		

3' portion	mt89-F *	CAGTACCTGCCCAGTGCGACAA	494 bp	58°C

	16SBR *	CCGGTCTGAACTCAGATCACGT		

full LSU rRNA	dCv-Ai-LSU-f	CTTTWGCAKMATGGCYTTWTGAG	~773 bp in *Cv*	55°C

	dCv-Ai-LSU-r	CACGGGGTCTTCTTGTCTWWCTTT	748 bp in *Ai*	

The final set of primers was used to amplify scallop (*Argopecten irradians*) genomic DNA and cDNA, and would amplify in *C. virginica *cDNA if the fragments were spliced post-transcriptionally.

### Secondary Structure Analysis of the Large Subunit rRNA

The LSU rRNA gene in each of the three respective cupped oyster mitochondrial genomes is split into two fragments separated by a large number of nucleotides (9958-9960 nts in *C. virginica*, 6662-6663 nts in *C. gigas*, and 11999 nts in *C. hongkongensis*); this phenomenon has not been previously characterized in any bilateral metazoan mitochondrial genome.

No introns were identified by screening the non-coding regions of the *C. virginica *mitochondrial genome using Rfam [48] and tools and data available at the **Comparative RNA Website (CRW) **[17]. Additional evidence that the two fragments of the LSU rRNA are not ligated into a single rRNA molecule are: (1) several complete protein genes (needed by the mitochondria for its function) are located between the two segments of the gene and have been observed in other mitochondrial genomes; (2) fragmentation in the oyster LSU rRNA gene occurs in highly variable region of the RNA, while introns, and the ligation of gene fragments, always occur (with a few minor exceptions) in highly conserved regions of the rRNA) [20]; and (3) the existence of expressed sequence tags (ESTs), not determined experimentally herein, revealed that the two fragments of the LSU rRNA were not ligated into a single RNA. We used these ESTs to infer the transcripts and gene boundaries. The 5' fragment of the *C. virginica *LSU rRNA is inferred to extend from the nucleotide immediately downstream from *trnD *(nt 8250, which is the extreme 5' position observed in the transcript data), to nucleotide position 8997, the site of polyadenylation in the majority of transcripts. The 3' fragment of the LSU rRNA gene in *C. virginica *is located from nt 1712 to nt 2430. The 5' boundary of this segment is based on two observations from transcript sequences: 1) nt 1712 is the 5' most position in ESTs matching the 3' portion of the LSU rRNA gene, and 2) nt 1711 is the polyadenylation site for transcripts containing the upstream cytochrome oxidase subunit 1 (*cox1*) gene. The right boundary is inferred from the observation that transcripts containing the 3' portion of the LSU rRNA gene are polyadenylated after position 2430. In *C. virginica *[GenBank: AY905542], eleven tRNA genes, and nine protein coding genes separate the 5' and 3' halves of the LSU rRNA gene [42].

In *C. gigas *[GenBank: AF177226], the inferred location of 5' fragment of the LSU rRNA gene is between nucleotides 5103 and 5703. Though we can not rule out overlapping gene-boundaries, the inferred start boundary of this fragment is at the first nucleotide following *trnQ*; nt 5117 represents the 5'-most position found in transcripts containing the LSU rRNA 5' section. The right boundary of this fragment is inferred by transcript polyadenylation at nt 5703/5704. The 3' fragment of the LSU rRNA gene is located between nucleotides 17265 and 17977 (slightly different from that annotated in [GenBank: AF177226 (17116..18224)]). The left boundary is based on the observation that *cox1 *transcripts in *C. gigas *extend to nt 17264, their polyadenylation site; the right boundary represents the polyadenylation site in transcripts containing the 3' portion of the LSU rRNA gene. Twelve tRNA genes, one SSU rRNA gene, nine protein coding genes, and the major non-coding region separate the 5' and 3' halves of the LSU rRNA gene [42].

In the *C. hongkongensis *mitochondrial genome by J. Ren and colleagues [44] [GenBank: EU672834], the 5' fragment of the LSU rRNA gene is located between nucleotides 7780 and 8384. The boundary of this fragment begins just downstream of *trnQ*. The mtDNA sequence by Yu et al. [43] [GenBank: EU266073] is incomplete and does not contain the *C. hongkongensis *5' fragment of the LSU rRNA gene sequence. The 3' fragment of the LSU rRNA gene is located between nucleotides 1761 and 2472 [GenBank: EU672834] according to Ren et al. [44], and similarly located at nucleotides 1764-2475 [GenBank: EU266073] by Yu et al. [43]; the two sequences [GenBank: EU672834 and EU266073] are 100% identical and 712 bp in length. Thirteen tRNA genes, one SSU rRNA gene, nine protein coding genes, and the major non-coding region separate the 5' and 3' halves of the LSU rRNA gene [44].

**Figure 2:**
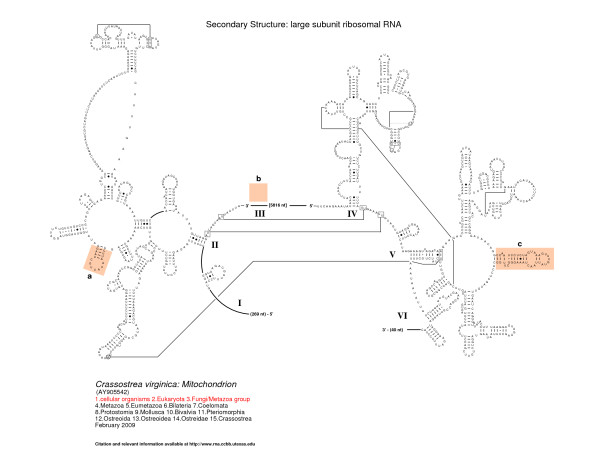
**Comparative secondary structure model of the LSU rRNA gene of *Crassostrea virginica***. Predicted locations of structural domains are represented by the roman numerals I - VI; however, Domain III is absent in the *C. virginica *LSU. Nucleotides in tertiary interactions that are predicted by comparative analysis are connected with a thinner line, while thicker lines reveal connections between consecutive nucleotides that are not immediately adjacent to one another. The fragmentation of the LSU rRNA occurs between the 3' end of structural domain II and the 5' start of domain IV. The three characteristic helical features considered in molluscan phylogeny are present in Domains II, III, and V and are highlighted as features *a, b*, and *c*, respectively [51]. As presented, feature *b *is absent in *C. virginica *LSU.

**Figure 3:**
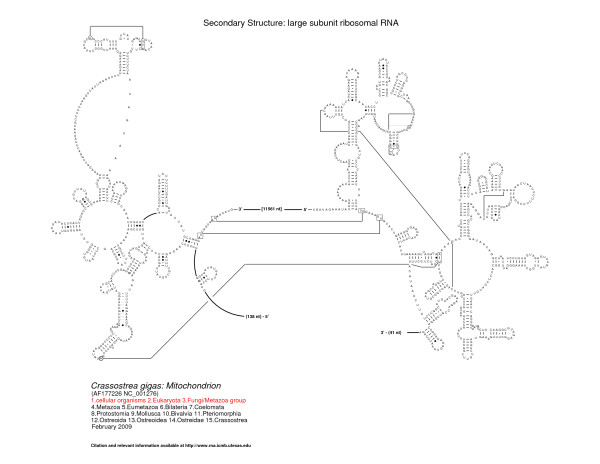
**Comparative secondary structure model of the LSU rRNA gene of *Crassostrea gigas***. Structural domains are represented by the roman numerals I - VI. Nucleotides in tertiary interactions that are predicted by comparative analysis are connected with a thinner line, while thicker lines reveal connections between consecutive nucleotides that are not immediately adjacent to one another. The fragmentation of the LSU rRNA occurs between the 3' end of structural domain II and the 5' start of domain IV.

**Figure 4:**
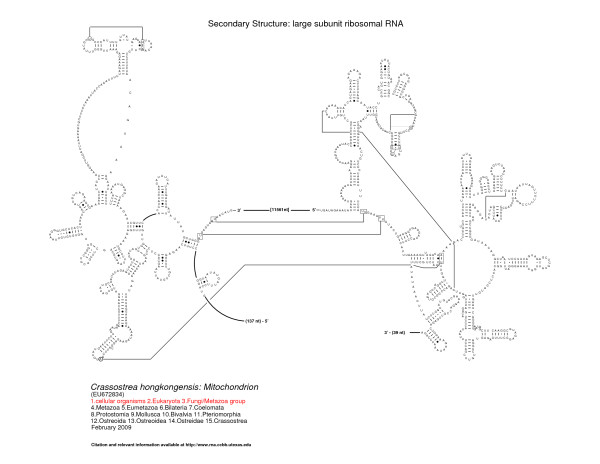
**Comparative secondary structure model of the LSU rRNA gene of *Crassostrea hongkongensis***. Structural domains are represented by the roman numerals I - VI. Nucleotides in tertiary interactions that are predicted by comparative analysis are connected with a thinner line, while thicker lines reveal connections between consecutive nucleotides that are not immediately adjacent to one another. The fragmentation of the LSU rRNA occurs between the 3' end of structural domain II and the 5' start of domain IV.

The 5' and 3' halves of the fragmented oyster LSU rRNA contain predicted secondary structural elements that are present in organisms spanning the entire tree of life [17,49-51], features characteristic of bilateral animal mitochondrial LSU rRNAs and features specific to molluscan LSU rRNA. Figures 2, 3, and 4 present the LSU rRNA secondary structures for *C. virginica, C. gigas*, and *C. hongkongensis*, respectively, and sequence similarities and characteristics are presented in Tables 2 and 3.

**Table 2:** Percent sequence similarity between *Crassostrea virginica *[42], *C. gigas *[42], and *C. hongkongensis *[43,44].

LSU *	*C. virginica*	*C. gigas*	*C. hongkongensis*
***C. virginica***	-	65	65

***C. gigas***	83	-	89

***C. hongkongensis***	84	96	-

* Similarities for the LSU 5' and LSU 3' are presented above and below the diagonal respectively.

**Table 3:** Summary of large subunit molecular characteristics for *Crassostrea virginica *[42], *C. gigas *[42], and *C. hongkongensis *[43,44].

	*C. virginica*	*C. gigas*	*C. hongkongensis*
**Large Subunit, 5'**			

Length (nt)	748	587	605

Percent A+T	66%	69%	68%

Percent G+C	34%	31%	32%

**Large Subunit, 3'**			

Length (nt)	719	713	712

Percent A+T	61%	61%	62%

Percent G+C	39%	39%	38%

In contrast to the 5' half of the molluscan LSU rRNA and other LSU rRNAs from organisms that span the tree of life, the 3' segment is more conserved among the molluscan rRNAs studied here and the 3' half of all LSU rRNA. The general conservation of the 5' and 3' halves of the molluscan LSU rRNA and all LSU rRNAs is consistent with the experimental studies that have associated more function to the 3' half than the 5' half. Although the oysters are closely related, interspecific sequence variation of the rRNAs is apparent (Table 2). The 5' half of the LSU rRNA is much more variable than the 3' half. The 3' LSU rRNA fragments in the three cupped oyster species have high nucleotide identity. *C. gigas *and *C. hongkongensis *are ~96% identical, while *C. virginica *exhibits a slightly lower similarity with *C. gigas *(82%) and *C. hongkongensis *(83%). Consistent with larger phylogenetic analyses, the 5' fragments exhibit much lower similarities between the species than that observed in the 3' fragments. The *C. virginica *5' fragment is only 65% identical with *C. gigas*. Yu et al. [43] do not report the 5' rRNA fragment, however it is included in GenBank: EU672834 by Ren et al. [44]. The *C. hongkongensis *5' fragment is 64% identical with that of *C. virginica *and 89% identical with *C. gigas*.

Consistent with other studies on molluscan rRNA genes [51], the A+T content is higher in the 5' versus 3' fragments (Table 3). The A+T content in the 5' LSU rRNA fragment is 66% in *C. virginica*, 69% in C. gigas, and 68% in *C. hongkongensis*; however, the A+T content in the 3' fragments of *C. virginica *and *C. gigas *is 61% and 62% in *C. hongkongensis*. The observed sequence similarities and phylogenetically consistent A+T content [51] between fragmented and continuous LSU rRNA genes implies that the same selective pressure is actively maintaining the functionality of the genes. Thus, it is unlikely that these fragments represent ancestral remnants or degenerate genes.

While the LSU rRNA domains present in all three oyster species reveal significant conservation with other metazoan mitochondria, there are distinct differences apparent when compared to rRNA structures of bacteria, archaea, and nuclear-encoded eukaryotes. Lydeard et al. [51] presented a detailed phylogenetic analysis of molluscan mitochondrial LSU rRNA secondary structures for a chiton, two bivalves, six gastropods, and a cephalopod. Mollusks generally exhibit all six of the typical LSU rRNA structural domains including three characteristic helical features that are critical in inferring the phylogeny of mollusks. The three characteristic helical features considered in molluscan phylogeny are present in Domains II, III, and V and are highlighted in Figure 2 as features *a, b*, and *c*, respectively.

Combining thorough analysis of these features, in addition to identifying fragmentation of mitochondrial LSU fragmentation in other molluscan bivalves may allow for a more accurate determination of phylogenetic relationships. A recent evaluation of the 3' half of the LSU rRNA secondary structure in the family Pectinidae (Mollusca: Bivalvia) revealed high levels of conservation in the secondary structure [52] with limited taxa differentiation. However, incorporation of fragmentation analysis within and between family level analyses may demonstrate a more acute phylogenetic representation of molluscan bivalves.

The currently discussed fragmentation is characteristic of just one branch of molluscan phylogeny, thus it is a general characteristic at this time and difficult to determine the diagnostic significance regarding phylogenetic relationships. However, as more molluscan mitochondrial genomes are investigated, and additional unique characteristics in the rRNA structure are discovered, such as this fragmentation and the features discussed above [51], it may become possible to specifically determine taxonomic branches. It may also become possible to differentiate the branch of the Mollusca based upon patterns of fragmentation. Additional molluscan mtDNA genomic sequences are necessary to determine the phylogenetic significance of these structural features (including the fragmentation).

The fragmentation of the LSU rRNA occurs between the 3' end of structural domain II and the 5' start of domain IV. Domain III is completely deleted; for comparison, domain III is composed of approximately 360 nucleotides in the bacterium *E. coli*. While its variation is limited in size and structure in the bacteria, archaea, and eukaryotes [17], fragmentation has been observed in different locations in domain III in a few eukaryotic nuclear-encoded rRNAs (for example, in the insect *Apis mellifera *[53]), and a few protists (such as *Euglena gracilis *[54]), and *Trypanosoma brucei *(X14553) [17]). Domain III is significantly truncated in size in bilateral animal mitochondria. Vertebrates typically maintain part of domain III, while some invertebrates such as *C. elegans *lack all, or nearly all, of domain III [17,55].

**Figure 5:**
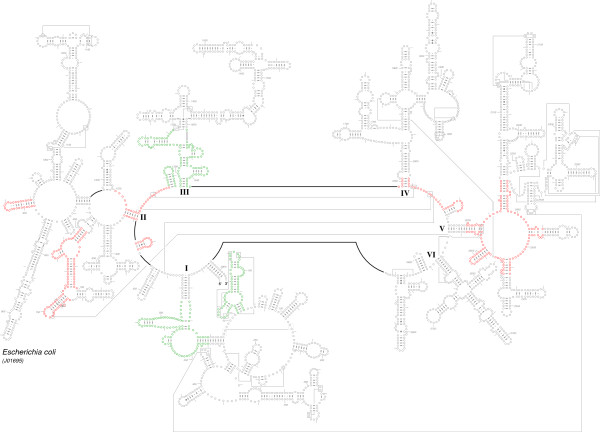
***Escherichia coli *23S rRNA **[17]. Regions that form the protein exit tunnel in the LSU ribosomal subunit are colored, red for the regions present in all of the LSU rRNAs that span the tree of life and green for the regions that are deleted in the oyster mitochondrial LSU rRNAs.

**Figure 6:**
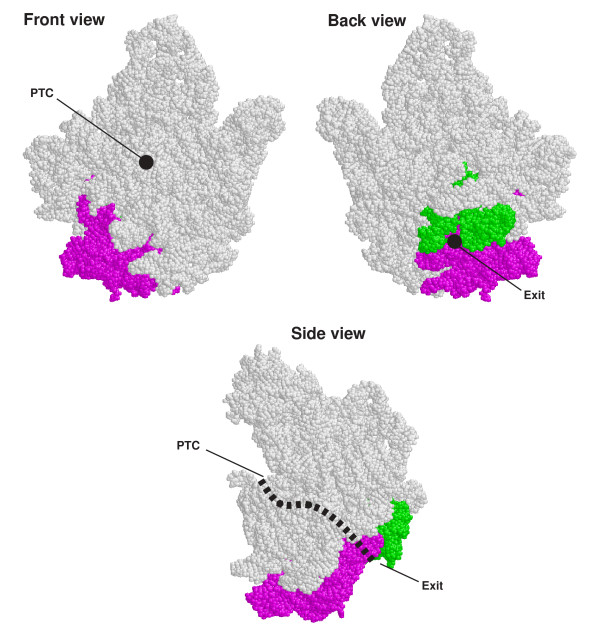
**Front, back, and views of the *Haloarcula marismortui *LSU rRNA high-resolution crystal structure**. While the approximate location of the peptide exit tunnel (PET) in the crystal structure is shown in a thick dotted line, the region of the LSU rRNA in domain I and III that are part of the protein exit tunnel in the *H. marismortui 50S subunit *and absent in the oyster mitochondrial LSU rRNAs are shown in green and purple (PDB ID 1JJ2)[58], respectively.

The peptide exit tunnel (PET) for growing proteins during protein synthesis in the 50S ribosomal subunit has been studied previously [56,57], which maps to the five domains of the LSU rRNA (Figure 5; red and green nucleotide positions are the regions associated with PET) and transverses through the 50S crystal structure (*Haloarcular marismortui*, PDB ID 1JJ2) [58]. The PET starts near the middle of this subunit at the peptidyl transferase center (PTC) and extends to the lower back of the 50S ribosomal subunit. The regions in domains II, IV, and V (red nucleotides) of the *H. marismortui *LSU rRNA associated with PET are present in all of the LSU rRNAs that span the tree of life while the regions in domains I and III (green nucleotides) are not present in the LSU rRNAs in the three *Crassostrea *mitochondrial genomes. In particular, domain III - immediately 3' to the end of the 5' LSU rRNA fragment and 5' to the start of the 3' LSU rRNA fragment - maps to the end of PET at the lower back of the *H. marismortui *50S ribosomal subunit (Figure 6; regions shown in purple). Thus the fragmentation of the oyster mitochondrial LSU rRNA occurs at one of the regions associated with PET that is deleted in these oyster LSU rRNAs. Interestingly, the deleted domains I and III in a cryo-EM structure of a mammalian mitochondrial ribosome were not replaced with ribosomal proteins nor with other portions of LSU rRNA [59]. Consequently, the absence of domains I and III (green and purple in Figure 6, respectively) shortens the exit tunnel by 25 Å compared to the bacterial PET of ~88 Å, suggesting that a growing peptide will be prematurely exposed to solvent as indicated by the cryo-EM structure. Currently it is not obvious why the fragmentation in the oyster LSU rRNAs occurs at the site directly involved in the formation of PET.

Nearly all of the other identified fragmentations of the rRNA genes contain a set of base pairings that form a helix that connects the two (or more) fragments of rRNA. Although no helix that is conserved in the three *Crassostrea *connect the two oyster LSU rRNA fragments, many tertiary interactions connect all regions of the LSU rRNA with one another in the high resolution crystal structures of the 50S ribosomal subunit (see the CRW links [17,60] which exhibit an interactive flash secondary structure browser and tables that reveal all of the secondary and tertiary interactions in the rRNA crystal structures, http://www.rna.ccbb.utexas.edu/SAE/2A/RNA2DMap/index.php, and http://www.rna.ccbb.utexas.edu/SAE/2A/xtal_Info/). Lastly, the ribosomal proteins interact with the rRNA and with one another to stabilize the network of interactions that provide the overall stability and dynamic behavior of the ribosome during protein synthesis. Thus the fragmentation in the rRNA, especially in a highly variable region, should not affect the structure nor the function of the ribosome since many organisms as noted in the introduction are fragmented and are functional.

Though several models have been developed, the evolutionary pathways responsible for fragmentation of mitochondrial ribosomal genes remain unknown. Several hypotheses have been developed for each organism in which fragmentation has been observed. For example, within just Chlamydomonads, it has been proposed that gene fragmentation has occurred by a bacterial-like transposition [28,61], recombination [28,29,62], horizontal transfer [63], acquisition and scrambling of processing sites [29], or it could be that the primordial ribosome was actually a noncovalent network of small RNA molecules [23,28]. As there are similarities in the degree of variability and regions that are excised, Schnare et al. [64] suggest that an ancestral ability to excise spacers within regions of variability may have been evolutionarily maintained within ciliates such as *T. pyriformis*. The evolutionary pathway that lead to oyster mtDNA LSU rRNA fragmentation is difficult to determine at this time due to the small number of fragmentations in the metazoans.

**Figure 7:**
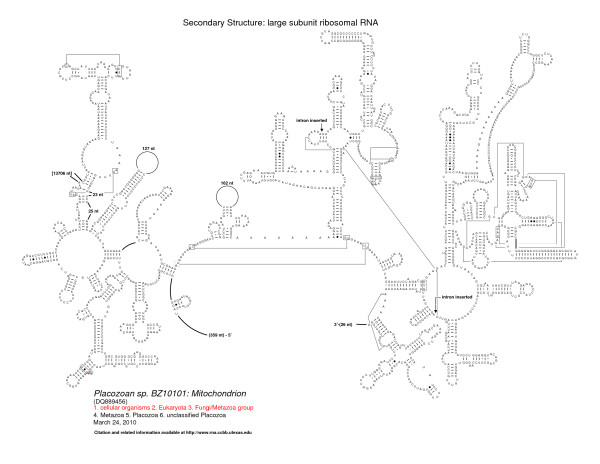
**Comparative secondary structure model of the LSU rRNA gene of the *Placozoan *species (BZ10101)**. Nucleotides in tertiary interactions that are predicted by comparative analysis are connected with a thinner line, while thicker lines reveal connections between consecutive nucleotides that are not immediately adjacent to one another. The position of the LSU rRNA fragmentation and the location of the group II intron are noted.

Discontinuous mitochondrial rRNA genes are rare in metazoans. Beyond the fragmented rRNA in oysters, the only other documented example of a fragmented mitochondrial rRNA is in the non-bilateral *Trichoplax adhaerens *and three other Placozoans [45,46,65] the simplest known free-living animal. For comparison with the oysters, we have generated secondary structures for these four Placozoan LSU rRNAs. We present one secondary structure here (Figure 7) and the remaining three are on the Comparative RNA Website). The LSU rRNAs from the Placozoans contain many of the structural elements present in Bacteria that are however absent in the bilateral animal LSU rRNAs. This contrasts with the bilateral animal mitochondrial LSU rRNAs which are highly truncated in comparison with the nuclear-encoded LSU rRNAs of Bacteria, Archaea, and Eukaryotes.

While the fragmentation and large truncation of the oyster mitochondrial LSU rRNA (that have been studied herein) occur between positions 1275-1650 (domains III and IV), all four Placozoan LSU rRNAs are fragmented after position 1013 (*Escherichia coli *numbering). This region of the rRNA is variable, consistent with a split in the rRNA without subsequent ligation. One of the Placozoan LSU rRNA structures (BZ10101) also had an insertion after position 1787, a region that is conserved in rRNAs that span the entire tree of life (including the mitochondria and chloroplast). This insertion was identified earlier [65] as a group II intron. Our analysis reveals that this intron appears to be in B subgroup of the group II introns. Two other introns occur after 1787. Both are mitochondrial, both are also group IIB, one is a green algae, *Pedinomonas minor *(AF116775) and the second is a brown algae, *Pylaiella littoralis *(Z48620). A third insertion in some of the Placozoan LSU rRNAs occurs after position 2586, which is in the peptidyl transferase loop, one of the most conserved regions in the rRNA. This intron, also identified previously [65] as a group II intron, also appears to be in the B subgroup. This is the first occurrence of an intron that we are aware of at this position. However many introns occur in the peptidyl transferase site, including at positions 2584 and 2585 http://www.rna.ccbb.utexas.edu/SAE/2C/rRNA_Introns/table1.php?show=condensed&from_gene = 23S[17,20].

The bilateral animals have on average about 16 kbp in their mitochondria. Their genomes are very compact, coding for approximately 37 genes that are in general truncated in size in comparison with Bacteria, Archaea, Eukaryotic nuclear-encoded and chloroplast, and with a minimal number of nucleotides not coding for protein or RNA between genes [66]. While the Cnidaria, Demospongiae, and Hexactinellida non-bilateral genomes are just slightly larger than the bilateral genomes (16 kbp - 25.6 kbp), the Placozoa non-bilateral genomes range from approximately 32 kbp - 43 kbp [66]. The Placozoan and a few of the other non-bilateral animals have multiple introns in their cytochrome oxidase subunit 1 and NADH dehydrogenase subunit 5 protein-coding genes, while none of the bilateral animal genomes have introns [46]. Phylogenetic analysis of the non-bilateral and bilateral Metazoan place the non-bilateral organisms on one branch and the bilateral on a separate major branch of the phylogenetic tree [46,67,68]. Although introns and trans-splicing have been observed in the Placozoan mitochondria, that have the largest animal mitochondrial genomes, we were not surprised that none were found (as discussed earlier) in the oyster genomes as their genome is typical in size and content with the other bilateral animal mitochondrial genomes.

Previous analysis of molluscan phylogeny has identified three particular mitochondrial LSU rRNA structural features that provide a phylogenetic signal [51]. The presence and/or absence of stem-loop structures within the LSU rRNA, as well length variation, previously resulted in a phylogenetic re-structuring of the molluscan taxa. Of the eleven mollusks (and *Drosophila melanogaster*) evaluated by Lydeard et al. [51], none of them exhibit the same characteristics as observed in the oysters and the fragmentation is unique. The phylogenic segregation from the predicted molluscan taxonomy warrant further examination and the fragmentation of the LSU rRNA gene in the oysters should be considered in future evaluation of molluscan phylogeny.

## Conclusions

Although many examples of discontinuous ribosomal genes have been documented in bacteria and archaea, as well as the nuclei, chloroplasts, and mitochondria of eukaryotes, oysters are the first characterized examples of fragmented bilateral animal mitochondrial rRNA genes. This study presents the secondary structure models of the discontinuous ribosomal RNA genes in the three cupped oyster (*Crassostrea*) mitochondrial genomes. Comparative structure models for the large subunit rRNA in each of the three oyster species are generally similar to those for other bilateral metazoans. While modeling the rRNA structure of the oyster, we determined that several of the rRNA secondary structure regions associated with the exit tunnel (identified in the high-resolution crystal structure of *Haloarcular marismortui*, PDB # 1JJ2) were in fact absent in the oyster mitochondrial LSU rRNA secondary structure. The biological and structural significance of this observation is not readily apparent.

To date the only other examples of discontinuous mitochondrial rRNA genes in Metazoans have been documented in Placozoans, which are considered to be the most primitive animals. We have generated comparative secondary structure models for several Placozoans LSU rRNAs (available at our supplemental site http://www.rna.ccbb.utexas.edu/SIM/4A/Fragmented_Mollusk/. While the fragmentation in the oyster and Placozoan mitochondrial LSU rRNA occurs in highly variable regions, consistent with other rRNA fragmentations, the specific location of the fragmentation is different between the species. Additionally, some Placozoans contain at least one group II intron. For the oysters involved in our studies, DNA sequence signatures, secondary structure analysis, RT-PCR experimentation, analysis of EST databases, and the absence of intron sequence and structural features all strongly suggest that these oyster mitochondrial rRNA fragments associate noncovalently to form a functional ribosome.

## Methods

### Reverse-transcriptase analysis

Ribonucleic acid (RNA) and deoxyribonucleic acid (DNA) were individually extracted from adductor muscle of the oyster *Crassostrea virginica *and the bay scallop *Argopecten irradians *using the RNeasy Mini kit (Qiagen) and the DNeasy Blood & Tissue kit (Qiagen), respectively, per manufacturer's instructions. Reverse-transcriptase (RT)-PCR was performed on the *C. virginica *and *A. irradians *RNA to generate complementary DNA (cDNA), using the High Capacity RNA-to-cDNA kit (Applied BioSystems Inc).

PCR amplification was performed to amplify portions of the 5' half and 3' halves of the LSU rRNA genes from both the DNA and cDNA templates. Three amplicons were investigated: 1) a 233 bp portion of the 5' half of the LSU rRNA gene, 2) and 434 bp portion of the 3' half of the rRNA LSU gene, and 3) a 748-773 bp amplicon that spans from the 5' portion into the 3' portion of the rRNA LSU gene. Primers for each of the three regions PCR amplicons are presented in Table 1. The third set of primers, though located in regions of conservation, are degenerate in order to amplify both *C. virginica *and *A. irradians *(GenBank: DQ665851). If the 5' and 3' fragments of the rRNA LSU are indeed splice post-transcriptionally, a ~773 bp amplicon will be amplified in the cDNA template of the oyster though not from its associated DNA template. The amplification of a 748 bp region of the fully contiguous LSU rRNA gene in *A. irradians *[47] in both genomic DNA and cDNA, serves as a positive control for the PCR reaction.

PCR was performed on an Eppendorf MasterCycler EP thermocycler using annealing temperatures defined in Table 1. The GoTaq polymerase system was employed with the following reagent conditions: 1X manufacturer supplied Flexi-PCR buffer (Promega Corporation), 1.5 mM MgCl2, 0.2 mM dNTPs, 0.2 μM primers, 5 U/μl GoTaq^® ^DNA polymerase (Promega Corporation). Approximately 50 ng of genomic DNA or 100 ng of cDNA template were incorporated in each reaction. PCR amplicons were visualized after agarose gel electrophoresis (with ethidium bromide staining). Final amplicons were Sanger sequenced to ensure amplification of the target region from both the DNA and cDNA template.

### Secondary Structure Modeling

The *C. virginica *mitochondrial genome [GenBank: AY905542][42] and that of *C. gigas *[GenBank: AF177226] were used to facilitate gene annotation and to generate putative secondary structures for each of the ribosomal genes. The recently published *C. hongkongensis *mitochondrial genomes from Yu et al. [43] [GenBank: EU266073] and Ren et al. [44] [GenBank: EU672834] were compared, particularly in regard to the rRNA gene content. EST collections for *C. virginica *and *C. gigas*, which contain numerous mitochondrial transcripts, were used to infer gene boundaries. EST databases were managed using SEQtools 8.1 [69]http://www.seqtools.dk.

The Comparative RNA Website and Rfam were employed to screen the mtDNA genome of the oyster for introns. Rfam [48] utilizes a nucleotide database (RFAMSEQ) of structural RNA sequences (including non-coding RNA genes and cis-regulatory RNA elements) to identify particular families of RNAs. Regions of the *C. virginica *mitochondrial genome denoted as non-coding regions were screened for introns that might mediate the splicing of the discontinuous LSU rRNA gene.

Secondary structure models were determined for the mitochondrial LSU rRNA genes of *C. virginica, C. gigas*, and *C. hongkongensis *using comparative structure models for LSU rRNAs from organisms that span the entire tree of life including animal mitochondrial rRNAs [17]. The prediction of several RNAs' secondary structure with comparative analysis is based on a very simple but profound concept. Each of the different types of RNA - e.g. tRNA, 5 S, SSU, and LSU rRNA form a similar secondary and three-dimensional structure, although the underlying primary structure, or sequence, can be significantly different.

Transfer RNA was the first molecule analyzed with this method once the first few tRNA sequences were determined in the early 1960's [70-74]. At the onset of these studies, a small number of sequences (e.g. tRNA) were inspected for similar helices that are present in the same region of the rRNA. The classic cloverleaf structure for the tRNA was determined to be that structure common to a growing number of tRNA sequences. The tRNA crystal structures [75,76] verified this cloverleaf secondary structure that was predicted with comparative methods.

This success with tRNA encouraged the use of comparative methods to predict other RNA secondary structures. The comparative structure for 5S rRNA was determined by Fox and Woese [77,78]. The prediction of the 16S and 23S rRNA secondary structures models with comparative analysis started after the first few bacterial sequences were determined in the early 1980's [79,80]. All of these early comparative studies on these RNA (and others not mentioned) were performed with a visual analysis of the RNA sequences for common structure. However, as the number of sequences increased, and larger sequences (e.g. 16S and 23S rRNA) were studied, these sequences were aligned for maximum sequence identity. Base pairs were predicted for those columns in an alignment that have similar patterns of variation, more commonly called covariation. In parallel, a series of computational methods were developed to identify and quantify the significance and purity of these covariations [17,18,81,82]. Initially we developed a simple number pattern method [18], followed by more sophisticated chi-square based methods [83] and mutual information [81,84] Many other comparative methods have been developed. A partial list is in Gardner and Giegerich [85]. Hidden Markov Models have also been used to predict RNA structure [86,87]. Other quantitative methods have been developed in the Gutell lab [17,82]. The determination of the proper alignment and secondary structure occur in parallel. Sequences in the alignment can be realigned once a common structural element is identified for a set of sequences that do not have sufficient sequence identity. Here these sequences are then aligned to maintain similarity in structure.

While an analysis of sequences from organisms that spanned the entire tree of life revealed parts of the rRNA that are conserved in primary and secondary structure, this analysis also revealed sequences and secondary structural elements that are only common to a subset of RNAs under study. These patterns of the variation and conservation in the rRNA are characteristic of an organism or group of organisms' location on the phylogenetic tree. The determination of the common secondary structure proceeds in stages. Each secondary structure model is considered a hypothesis that is tested with each new sequence. As the number and diversity in the sequences increases, the structure models are modified. Some previously proposed base pairings are removed when both positions of a predicted base pair do not have a similar pattern of variation while new base pairs are predicted relative to the increase in the number and variation in sequences when analyzed with improved, more sensitive covariation algorithms. Every secondary structure model is not determined *de novo*; instead every new secondary structure we model with comparative analysis is derived from the previously proposed secondary structures that are the most similar in sequence. Thus, as this determination of secondary structures proceeds from the root of the tree to the outer branches, the newly determined secondary structure has less and less variation from the template structure it is derived from. The **Comparative RNA Website (CRW) **[17], http://www.rna.ccbb.utexas.edu contains more than 1000 rRNA secondary structure diagrams. More secondary structure diagrams are continually determined to increase the sampling of organisms that span the entire tree of life, thus capturing more of the variations that are usually diagnostic of a group of related organisms. Molluscan rRNA structures determined previous [51] were used to derive the comparative structure models of the oyster mitochondrial rRNAs. Additional details for the methods and predicted structure models are available at the **CRW **http://www.rna.ccbb.utexas.edu/, http://www.rna.ccbb.utexas.edu/CAR/1D/; the CRW contains supplemental information http://www.rna.ccbb.utexas.edu/SIM/4A/Fragmented_Mollusk/.

## Authors' contributions

CAM carried out the molecular genetic assays, participated in the sequence annotation, and drafted the manuscript. JJC and JCL modeled the secondary rRNA structures and contributed to the preparation of the manuscript. PMG has made substantial contributions to project design, analysis and interpretation of data, and revision of this manuscript. RRG oversaw the secondary structure modeling and contributed to the preparation of the manuscript. All authors read and approved the final manuscript.
